# The promise of IL-1β modulation in NSCLC clinical context

**DOI:** 10.3389/fimmu.2026.1773253

**Published:** 2026-01-30

**Authors:** François Ghiringhelli, Cédric Rébé

**Affiliations:** Université Bourgogne Europe, Centre Georges-François Leclerc, Unicancer, Cancer Biology Transfer Platform, UMR INSERM 1231, TIRECs Team, Equipe Labellisée LIGUE 2024, Dijon, France

**Keywords:** chemotherapy, clinical trials, IL-1β, immunotherapy, NSCLC

## Abstract

Emerging preclinical evidence challenges the long-standing assumption that Interleukin-1β (IL-1β) uniformly promotes non–small cell lung cancer (NSCLC). We show that, in the context of chemo-immunotherapy, IL-1β enhances anti-tumor immunity by inducing tumor-cell CXCL10 expression and recruiting CD8^+^ T cells, thereby sensitizing “cold” tumors to treatment. These findings contrast sharply with the failure of multiple CANOPY trials targeting IL-1β, suggesting that blockade may be effective only in prevention or early carcinogenesis. Instead, controlled IL-1β activation, guided by biomarkers and combined with chemotherapy plus PD-1 blockade, may represent a promising strategy to overcome resistance in established NSCLC.

## Introduction

We recently published a preclinical work providing a compelling re-interpretation of the role of Interleukin-1β (IL-1β) in non-small cell lung cancer (NSCLC), demonstrating that, in the context of chemo-immunotherapy, IL-1β can significantly enhance anti-tumor immune responses. By promoting tumor-cell intrinsic expression of the chemokine CXCL10 (the key T cell chemoattractant molecule), IL-1β drives the recruitment of CD8^+^ T cells, overcoming resistance to standard platinum-based chemotherapy plus anti-PD-1, in “cold tumors” (1). The implication is provocative: rather than inhibiting IL-1β, inducing its activity (or enabling its production) might sensitize “cold tumors” to immunotherapy.

This paradigm sharply contrasts with the prevailing clinical efforts over the past several years, which have focused almost exclusively on blocking IL-1β. The rationale for blockade came from preclinical studies (2–4) and also from epidemiological and early experimental evidence that IL-1β promotes tumorigenesis, notably the findings from the cardiovascular CANTOS trial, where patients receiving Canakinumab (a monoclonal anti-IL-1β antibody) had reduced incidence and mortality of lung cancer (5). The observation that air pollutants cause the release of IL-1β by lung macrophages, thus resulting in the proliferation of progenitor-like *EGFR* mutant epithelial cells, strengthened the pro-tumoral action of IL-1β (6).

That led to a wave of trials (the CANOPY program) in NSCLC: first-line, adjuvant, neoadjuvant, and second-line settings. Yet, frustratingly, the vast clinical promise of IL-1β blockade has not materialized so far (7–10).

## Clinical trial outcomes: disappointing results with IL-1β blockade

In the first-line setting, the Phase III trial CANOPY-1 (NCT03631199) combined Canakinumab with pembrolizumab + platinum-based chemotherapy in advanced/metastatic NSCLC. The study did not meet its primary endpoints: median progression-free survival (PFS) was identical between Canakinumab and placebo arms (6.8 months, hazard ratio [HR], 0.85; 95% CI, 0.67 to 1.09; *P* = 0.102), and overall survival (OS) was not significantly improved (20.8 months vs 20.2 months, HR, 0.87; 95% CI, 0.70 to 1.10; *P* = 0.123) (7).

In the adjuvant setting, the Phase III trial CANOPY-A (NCT03447769) tested Canakinumab after complete surgical resection and cisplatin-based chemotherapy in stage II/III NSCLC. It failed to demonstrate a statistically relevant benefit in disease-free survival (DFS): median DFS was 35.0 months with Canakinumab vs 29.7 months for placebo (hazard ratio 0.94; 95% CI, 0.78 to 1.14; one-sided *P* = 0.258) (8).

In earlier-stage, neoadjuvant treatment, the Phase II trial CANOPY-N (NCT03968419) evaluated Canakinumab alone or in combination with pembrolizumab before surgery. The major pathological response (MPR) rate was low and did not significantly improve over pembrolizumab alone (10).

A prior third-line trial, CANOPY-2 (NCT03626545), combining Canakinumab with docetaxel in patients whose disease progressed after chemotherapy and PD-(L)1 therapy, also failed to meet its OS endpoint: median 10.6 months (95% confidence interval [CI], 8.2–12.4) for the canakinumab arm and 11.3 months (95% CI, 8.5–13.8) for the placebo arm (hazard ratio, 1.06 [95% CI, 0.76–1.48]; one-sided *P-*value = 0.633) (9).

Thus, despite a sound mechanistic rationale and strong epidemiological signals, anti-IL-1β therapy has so far shown no consistent clinical benefit in NSCLC across multiple settings, first-line metastatic, adjuvant, neoadjuvant, or salvage therapy. In a preventive setting, many clinical trials are currently evaluating the effects of canakinumab, including in smoker populations (NCT04789681, NCT05725343, NCT06038526).

## Reconciling preclinical promise with clinical failure - what went wrong?

**Figure 1:**
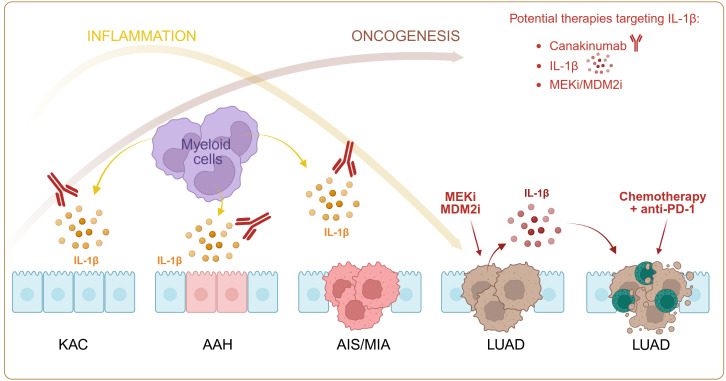
Role of IL-1β in the chronology of lung cancer development. In early stages (from KAC to AIS/MIA), IL-1β participates in tumor generation and progression. Its blockade may hinder oncogenesis. In late stages (LUAD), IL-1β or inducers (MEKi and MDM2i) may overcome resistance to chemo-immunotherapy, by triggering CD8 T cell recruitment at tumor site. In red, proposed therapeutical tools. AAH, Atypical Adenomatous Hyperplasia; AIS, Adenocarcinoma *In Situ;* KAC, *KRT8* high Alveolar intermediate Cell; LUAD, LUng ADenocarcinoma; MDM2i, Murine Double Minute 2; MEKi, Mitogen-activated protein Kinase kinase; MIA, Minimally Invasive Adenocarcinoma. Figure made with Biorender.

The discrepancy between our optimistic preclinical data and discouraging clinical outcomes (the failure of Canakinumab in the CANOPY trials) may be explained by the timing and context matters. Our preclinical data suggest that IL-1β helps activate immune responses when combined with chemotherapy + Immune Checkpoint Inhibitors (ICIs) and turns “cold tumors” into “hot” ones. In contrast, the CANOPY trials attempted to use IL-1β blockade broadly, regardless of immune and stage contexts. IL-1β may still contribute to protumor inflammation (angiogenesis, invasiveness) (6). In *KRAS*-mutant cases, *IL1B-IL1R1* interactions were markedly more prevalent in precursor lesions (100%) than in invasive lesions. These findings highlight the role of IL-1β-IL1-R1 axis and proinflammatory cues in driving oncogenesis of KACs (*KRT8* high alveolar intermediate cell) during early LUAD pathogenesis (11). Thus, early inflammatory events drive carcinogenesis, but once a tumor is established, the same cytokine might adopt a different role, more particularly in the right conditions, e.g. under chemo-immunotherapy treatments (**Figure 1**). Thus, the therapeutic window for IL-1β inhibition may be limited to cancer prevention rather than treatment.

## How the new preclinical findings could reshape clinical strategies

The mechanistic insights from our study prompt to reconsider IL-1β not as a monolithic “bad actor” but as a context-dependent modulator of anti-tumor immunity. Clinically, this suggests a shift in strategy rather than abandonment of IL-1β-targeted therapies. Here are some potentially transformative clinical perspectives:

### Refined use of IL-1β blockade - prevention or specific subgroups

The failure of CANOPY to improve survival does not entirely rule out IL-1β blockade in all contexts. Instead, it suggests that its use should likely remain in prevention (high-risk individuals) or in specific patient subsets with chronic inflammatory signatures driving tumorigenesis. For example, former heavy-smokers or people with chronic pulmonary inflammation might still benefit from IL-1β neutralization before overt cancer emerges.

### Testing IL-1β “induction” strategies in combination with chemo-immunotherapy

Chronic or systemic activation of IL-1β may provoke inflammation-related toxicity (fever, systemic inflammatory responses) or worsen comorbidities. In our preclinical experiments, we already note toxicity (e.g., weight loss in mice) associated with recombinant IL-1β administration (1). This observation led us to investigate the potential of molecules that can trigger IL-1β production.

Based on our study’s demonstration that artificially boosting IL-1β or activating its pathway sensitizes tumors to chemotherapy + anti-PD-1, clinical trials might explore agents that provoke inflammasome activation in tumor cells. Inflammasomes are multiproteic intracellular platform that trigger caspase-1 activation a protease responsible for inactive pro-IL-1β maturation into active IL-1β (12). For example, combining standard first-line chemo-immunotherapy with safe “inflammasome inducers” (small molecules, drugs modulating mitochondrial stress) might convert otherwise resistant tumors into responders. In this setting, we propose several candidate drugs inducing the IL-1 pathway at the tumor site, like trametinib (MEK inhibitor) or JNJ-26854165 (MDM2 inhibitor) that reverse resistance to chemo-immunotherapy, as shown in experimental models (1) (**Figure 1**).

### Biomarker-driven treatment indications

Rather than administering immunotherapy with or without chemotherapy, future clinical practices should stratify patients based on tumor-intrinsic and microenvironment biomarkers, like *IL1B* and/or *IL1R1*. High tumor expression of IL-1 pathway genes (*IL1B, IL1R1*) correlates with better PFS and OS in immunotherapy-treated patients, but not in those receiving chemotherapy alone, thus suggesting that such signature can be used as a predictive marker (1). In the same way, the use of IL-1β-inducing therapies could be conditioned to a high expression of the molecular targets responsible for IL-1β maturation and production.

## Conclusion

The failure of Canakinumab in the CANOPY trials was disappointing, but perhaps not surprising in retrospect: those trials were built on the assumption that IL-1β uniformly promotes cancer, and that its inhibition would therefore be beneficial across NSCLC. Our new preclinical data suggest a far more complex reality: IL-1β may not always be the enemy, sometimes, it’s the ally immunotherapy needs.

Translating this insight into the clinic will require a radical shift: from inhibition to precision activation, guided by biomarkers, delivered in a controlled and temporal manner, and integrated into standard-of-care chemo-immunotherapy regimens. Given the burden and poor prognosis of NSCLC, such an effort is more than justified.

In that light, the current state of IL-1β-targeted therapy should not be seen as the end of the story, but rather as the prelude to a new chapter, where controlled modulation of innate immunity may unlock durable responses for patients who currently have few options. From now, the question to block or to induce IL-1β in lung cancer should be addressed and clarified in further clinical trials.

## Data Availability

The original contributions presented in the study are included in the article/supplementary material, further inquiries can be directed to the corresponding author/s.

## References

[1] Perrichet A Lecuelle J Limagne E Thiefin M Bellio H Jacob P . Cancer cell-derived IL-1β reverses chemo-immunotherapy resistance in non-small cell lung cancer. Nat Commun. (2025) 16:10244. doi: 10.1038/s41467-025-64839-4, PMID: 41271647 PMC12639146

[2] Wang L Zhang L-F Wu J Xu S-J Xu Y-Y Li D . IL-1β-mediated repression of microRNA-101 is crucial for inflammation-promoted lung tumorigenesis. Cancer Res. (2014) 74:4720–30. doi: 10.1158/0008-5472.CAN-14-0960, PMID: 24958470

[3] Huang J Lan X Wang T Lu H Cao M Yan S . Targeting the IL-1β/EHD1/TUBB3 axis overcomes resistance to EGFR-TKI in NSCLC. Oncogene. (2020) 39:1739–55. doi: 10.1038/s41388-019-1099-5, PMID: 31740781

[4] Yuan B Clowers MJ Velasco WV Peng S Peng Q Shi Y . Targeting IL-1β as an immunopreventive and therapeutic modality for *K-ras*–mutant lung cancer. JCI Insight. (2022) 7:e157788. doi: 10.1172/jci.insight.157788, PMID: 35471938 PMC9220853

[5] Ridker PM MacFadyen JG Thuren T Everett BM Libby P Glynn RJ . Effect of interleukin-1β inhibition with canakinumab on incident lung cancer in patients with atherosclerosis: exploratory results from a randomised, double-blind, placebo-controlled trial. Lancet. (2017) 390:1833–42. doi: 10.1016/S0140-6736(17)32247-X, PMID: 28855077

[6] Hill W Lim EL Weeden CE Lee C Augustine M Chen K . Lung adenocarcinoma promotion by air pollutants. Nature. (2023) 616:159–67. doi: 10.1038/s41586-023-05874-3, PMID: 37020004 PMC7614604

[7] Tan DSW Felip E de Castro G Solomon BJ Greystoke A Cho BC . Canakinumab versus placebo in combination with first-line pembrolizumab plus chemotherapy for advanced non–small-cell lung cancer: results from the CANOPY-1 trial. J Clin Oncol. (2024) 42:192–204. doi: 10.1200/JCO.23.00980, PMID: 38039427

[8] Garon EB Lu S Goto Y De Marchi P Paz-Ares L Spigel DR . Canakinumab as adjuvant therapy in patients with completely resected non-small-cell lung cancer: results from the CANOPY-A double-blind, randomized clinical trial. J Clin Oncol. (2024) 42:180–91. doi: 10.1200/JCO.23.00910, PMID: 37788412

[9] Paz-Ares L Goto Y Wan-Teck Lim D Halmos B Chul Cho B Cobo M . Canakinumab in combination with docetaxel compared with docetaxel alone for the treatment of advanced non-small cell lung cancer following platinum-based doublet chemotherapy and immunotherapy (CANOPY-2): A multicenter, randomized, double-blind, phase 3 trial. Lung Cancer. (2024) 189:107451. doi: 10.1016/j.lungcan.2023.107451, PMID: 38354535

[10] Lee JM Pujol J-L Zhang J Leonov O Tsuboi M Kim ES . CANOPY-N: A phase 2 study of canakinumab or pembrolizumab, alone or in combination, as neoadjuvant therapy in patients with resectable, stage IB–IIIA NSCLC. JTO Clin Res Rep. (2025) 6:100859. doi: 10.1016/j.jtocrr.2025.100859, PMID: 40810132 PMC12343357

[11] Peng F Sinjab A Dai Y Treekitkarnmongkol W Yang S Bolanos LIG . Multimodal spatial-omics reveal co-evolution of alveolar progenitors and proinflammatory niches in progression of lung precursor lesions. Cancer Cell. (2025) 44:1–19. doi: 10.1016/j.ccell.2025.10.004, PMID: 41202811 PMC12980502

[12] Rébé C Ghiringhelli F . Interleukin-1β and cancer immune response. Semin Immunol. (2025) 80:102002. doi: 10.1016/j.smim.2025.102002, PMID: 41176853

